# Case report: Cancer-free survival after chemotherapy, targeted immunotherapy combination with proton therapy following space making technique in a patient with cholangiocarcinoma after choledochal cyst resection

**DOI:** 10.3389/fimmu.2024.1520248

**Published:** 2025-01-08

**Authors:** Jian Kong, Qi Xia, Li Xu, Dongcun Jin, Wenbing Sun

**Affiliations:** ^1^ Department of Hepatobiliary-Pancreatic-Splenic Surgery, Beijing Chaoyang Hospital, Capital Medical University, Beijing, China; ^2^ Department of Hepatobiliary-Pancreatic-Splenic Surgery, Chaoyang Central Hospital, Chaoyang, Liaoning, China; ^3^ Proton Therapy Center, Tsuyama Chuo Hospital, Tsuyama, Okayama, Japan

**Keywords:** choledochal cyst, cholangiocarcinoma, space making, proton therapy, immunotherapy

## Abstract

Choledochal cysts (CCs) are rare cystic dilations of the intrahepatic and/or extrahepatic bile ducts. Malignancies arising during follow-up after excision of CCs have been reported in both children and adults, with no typical time frame for malignancy development. We present a case of a patient diagnosed with CCs 36 years ago, who underwent resection and subsequently developed cancer. The patient received chemotherapy, targeted therapy, and immunotherapy, with efficacy evaluation indicating a state of stable disease. Considering tumor resistance after continuous systemic therapy and an unresectable tumor, proton therapy was selected for the next treatment. To prevent gastrointestinal side effects after proton therapy, the bile-enteric anastomosis was dismantled, and a greater omentum strip was used to fill the subhepatic space, creating a barrier between the biliary duct and the intestine. The patient successfully underwent proton therapy without any gastrointestinal complications. As CC-associated malignancy poses a lifelong risk even with complete resection, surveillance should be maintained throughout the follow-up period. Comprehensive treatment should be adopted to improve prognosis in malignancy after CC resection.

## Introduction

Choledochal cysts (CCs) are rare, cystic dilations of the intrahepatic and/or extrahepatic bile ducts (1). Approximately 80% of CCs are diagnosed in childhood, often presenting with a right upper quadrant mass, abdominal pain, and jaundice (1, 2). CCs can cause complications such as recurrent cholangitis, acute pancreatitis, secondary biliary cirrhosis, and a significant lifetime risk of malignancy. Surgery is recommended to prevent long-term complications of CCs, even in asymptomatic patients (2). However, malignancies have been reported during follow-up after CC excision in both children and adults, with no typical time frame for malignancy development (3). We report a case of a patient diagnosed with CCs 36 years ago, who underwent surgical resection and subsequently developed cancer. The patient received comprehensive treatment, including chemotherapy, targeted therapy, immunotherapy, and proton therapy. To prevent side effects after proton therapy, we applied a spacing technique to create a space to isolate the bile duct and the intestine.

## Case description

A female patient was hospitalized in 1988 due to obstructive jaundice and was diagnosed with CCs of Todani type I. Cholecystectomy and choledochal cyst jejunal anastomosis were performed. In 2005, the patient was admitted to our department with upper abdominal pain and was diagnosed with a congenital bile duct cyst. CCs resection and cholangiojejunostomy were performed. The pathological diagnosis was congenital cystic dilation of the bile duct with clean ends. Regular follow-ups from 2005 to January 2023 revealed no obvious abnormalities. She was admitted to the hospital in January 2023 due to fever, chills, and jaundice of the skin and sclera. On physical examination, surgical scars were visible on the upper abdomen, the abdomen was soft, upper abdominal tenderness was absent, and there was no rebound tenderness or muscle tension. Her initial laboratory results showed a white cell count of 5.6×10^9/L, hemoglobin of 113 g/L, and a platelet count of 257×10^9/L. Liver function tests showed TBIL of 50 μmol/L, DBIL of 42.8 μmol/L, ALT of 206 U/L, AST of 135 U/L, and ALP of 932 U/L. Tumor markers such as CA-199 >1000.00 IU/ml, CA-242 >300.00 U/mL, and CA-50 >180.00 U/mL were significantly elevated. The level of immunoglobulin G1-4 was normal.

**Figure 1:**
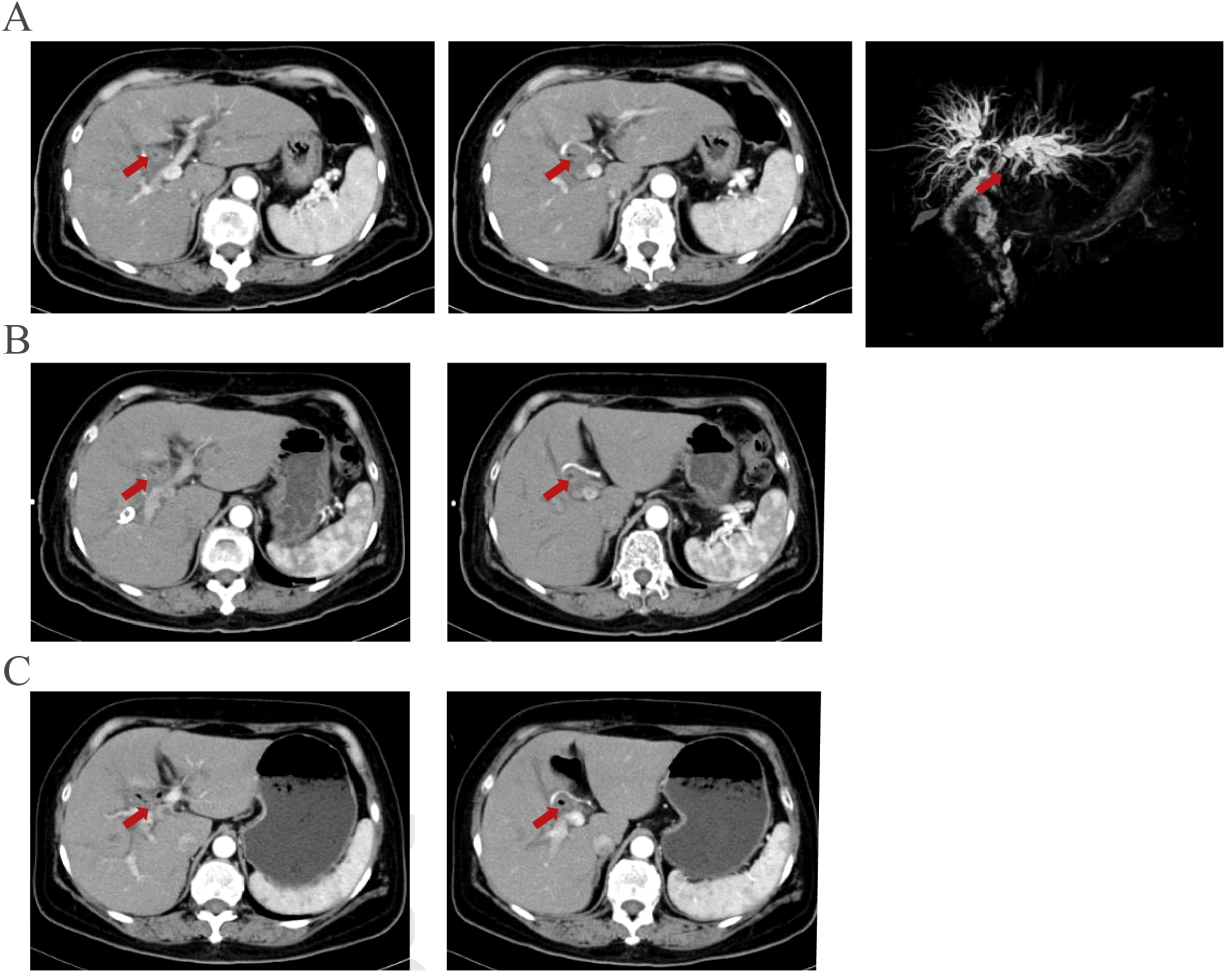
Imaging manifestations before and after systemic therapy were demonstrated. **(A)** An enhanced lesion is located at the bile duct near the choledochojejunostomy site in the arterial phase of contrast-enhanced computed tomography and MRCP before PTCD and chemotherapy. **(B)** The enhanced lesion is located at the bile duct near the choledochojejunostomy site showed no significant changes after PTCD and therapy plus durvalumab for three cycles. **(C)** The lesion showed no significant changes after three more therapy plus durvalumab and bevacizumab.

**Figure 2:**
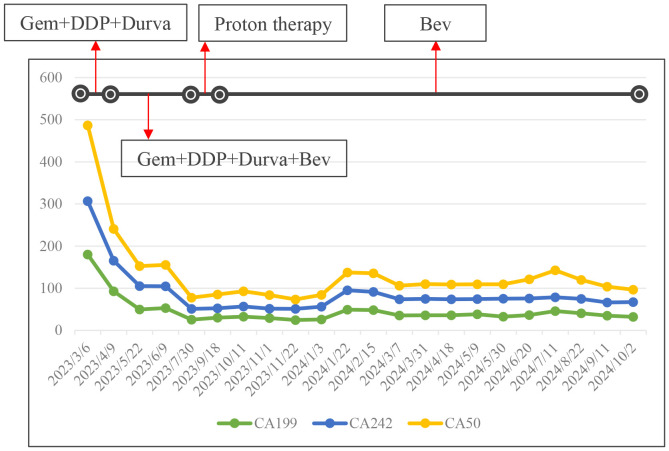
The trend change of CA199, CA242 and CA50 were demonstrated at different stages of treatment. Gem, gemcitabine; DDP, cisplatinum; Durva, druvalumab; Bev, bevacizumab.

Abdominal enhanced CT, MRI, and MRCP showed hilar cholangiocarcinoma and intrahepatic bile duct dilation (**Figure 1A**). The initial diagnosis was hilar cholangiocarcinoma and obstructive jaundice. Percutaneous transhepatic cholangial drainage (PTCD) and gemcitabine plus cisplatin chemotherapy for one cycle were performed. The patient tolerated gemcitabine plus cisplatin chemotherapy well and was treated with gemcitabine plus cisplatin chemotherapy followed by durvalumab for two cycles. After PTCD, bilirubin and transaminase levels gradually returned to normal. There were no abnormalities after the drainage tube was elevated and clamped, leading to the removal of the PTCD. CA199, CA242, and CA50 levels gradually decreased after systemic therapy (**Figure 2**). Abdominal imaging demonstrated minimal tumor shrinkage. Therefore, bevacizumab was added to the original chemotherapy regimen for three cycles. CA199, CA242, and CA50 levels gradually returned to normal (**Figure 2**). Re-imaging evaluation revealed minimal tumor shrinkage (**Figures 1B, C**).

**Figure 3:**
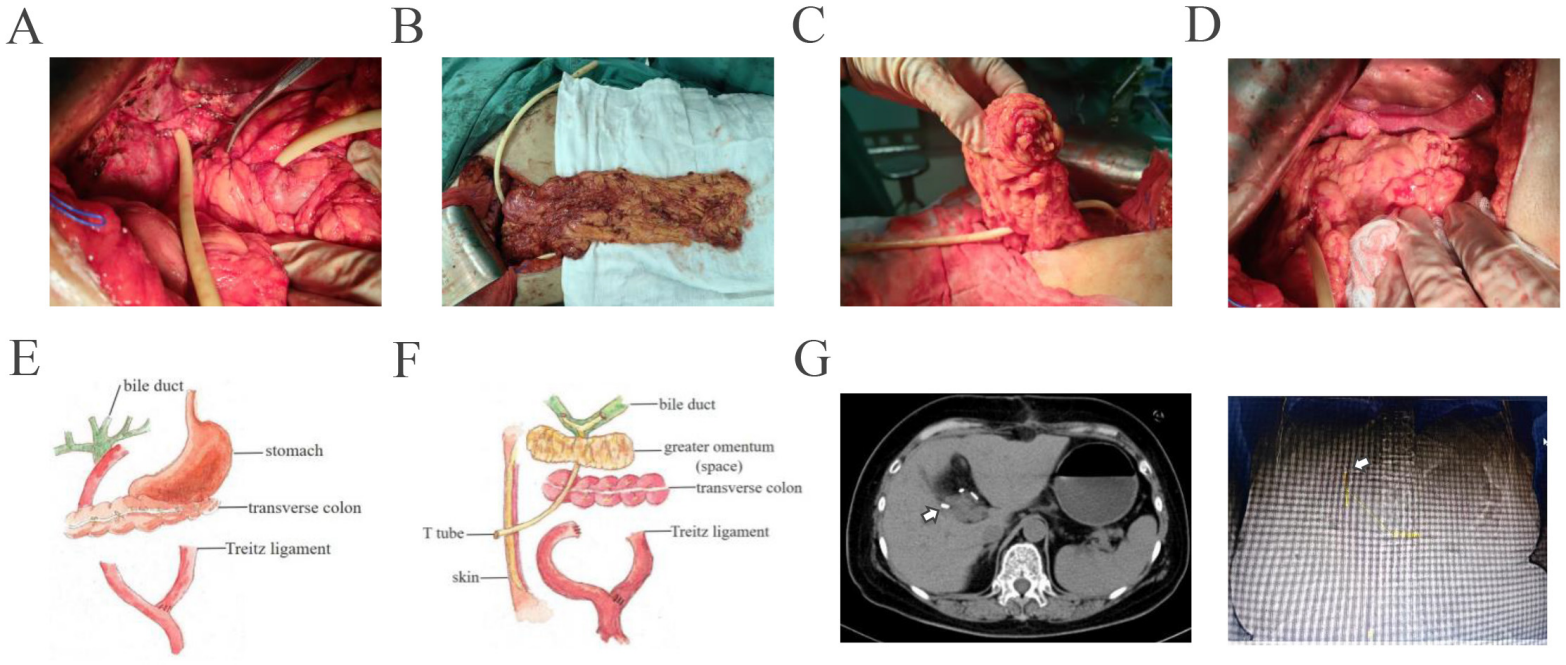
The procedures of space making between biliary tract and intestinal tract were shown. **(A)** Dismantle the bilioenteric anastomosis, perform T-tube support and drain the left and right hepatic ducts. **(B)** Free partial greater omentum in strip-type. **(C)** Roll up the distal 1/2 of the greater omentum. **(D)** Fill the subhepatic space with the greater omentum. Move the gastric antrum, duodenal bulb, and transverse colon away from the porta hepatis. **(E, F)**. Schematic diagram shows preoperative and postoperative conditions of the patient. **(G)** Titanium clips were placed around the left and right hepatic ducts for easy of location of proton therapy. White arrow indicated the location of titanium clips.

**Figure 4:**
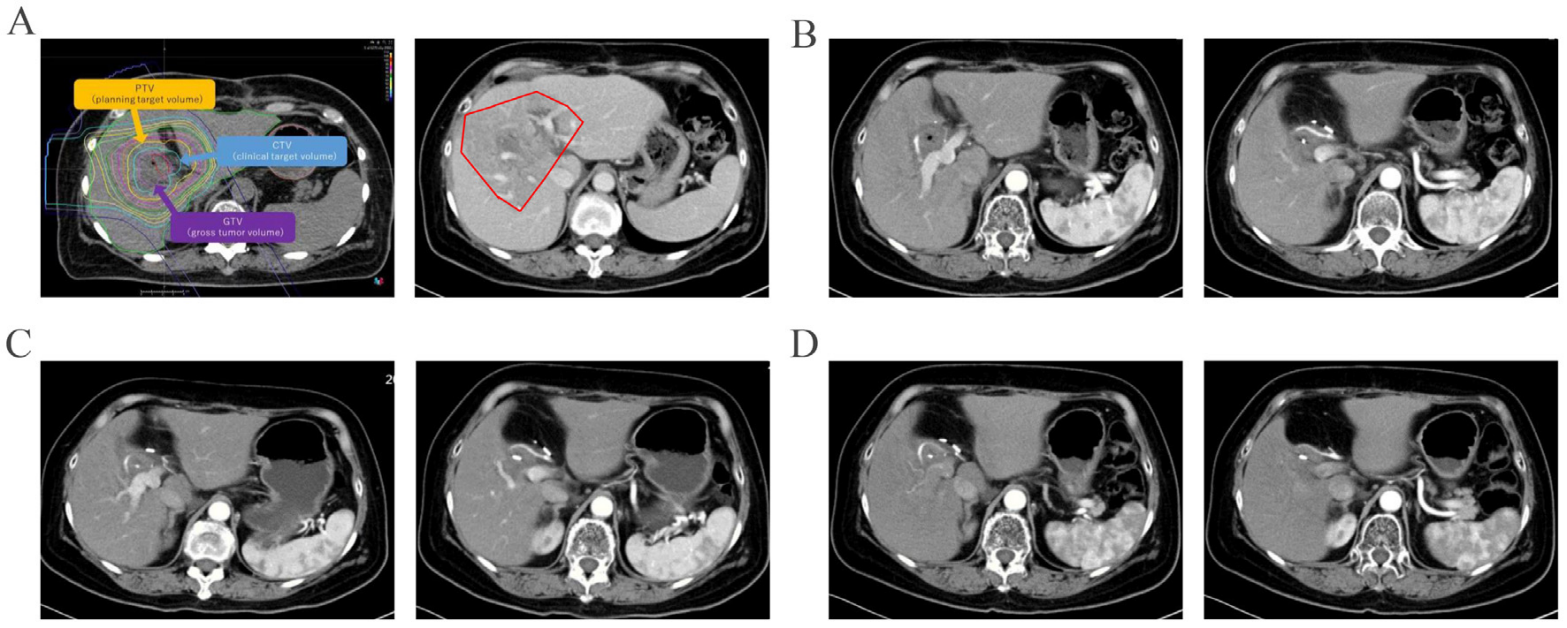
Imaging follow-up after proton therapy were shown. The irradiation field of proton therapy was showed before and after therapy **(A)**. And original lesion site did not show enhancement in the arterial phase three months after proton therapy **(B)**. The original lesion site did not show enhancement in the arterial phase seven months **(C)** and ten months **(D)** after proton therapy.

Given the potential for tumor resistance after chemotherapy and the unresectable nature of the tumor, surgical resection was not considered. Proton therapy was considered for the next step of treatment. To mitigate the adverse effects of proton therapy on the gastrointestinal tract, space-making surgery was performed (**Figure 3**). A space of at least 2 cm was created between the digestive tract and the tumor to block radiation. The bile-enteric anastomosis was dismantled, and T-tube support and drainage of the left and right hepatic ducts were placed (**Figure 3A**). A partial greater omentum strip was dissected, rolled up, and used to fill the subhepatic space, creating a barrier between the biliary duct and the intestine (**Figures 3B–F**). Titanium clips were placed around the left and right hepatic ducts to facilitate photon therapy localization (**Figure 3G**). Proton therapy was performed with a radiation dose of 62.7GyE/19 fractions. The patient did not experience any gastrointestinal-related complications. Durvalumab was used for subsequent maintenance therapy. Regular follow-up indicated that the patient is currently in a disease-free survival state, with normal levels of tumor markers and no signs of tumor recurrence detected (**Figures 2**, **4**).

## Discussion

Malignancies arising during follow-up after CC excision have been reported in both children and adults, with no typical time frame for malignancy development. Once malignancy occurs, the prognosis is poor, and the principles of treatment should be the same as those for biliary tract cancer. In the present case, the patient underwent various comprehensive treatments and is currently in a disease-free survival state.

The incidence of CCs is broadly reported across the literature as occurring in 1/100,000 to 1/150,000 in Western populations and as high as 1/1000–1/13,000 in East Asian populations (3, 4). The occurrence of biliary tract cancer during follow-up after CC excision can be as high as 11.4% in adults over the age of 18 years, with a median age for diagnosis of 42 years (5). Cases have been identified as malignancy after short (up to 5 years), mid-term (up to 10 years), or long-term (up to 34 years) follow-ups (6). Cholangiocarcinoma is the most frequently reported malignancy, followed by adenocarcinoma of the gallbladder and pancreas (4). An increase with age reflects the increased risk for biliary tract cancer in CC excision cases, and the cancer risk seems reduced but not eliminated with complete resection (5, 6). In our case, hilar cholangiocarcinoma occurred 36 years after CC excision, highlighting the importance of regular follow-up.

Although many articles have commented on the poor prognosis of cholangiocarcinoma, information on the management of CC-associated malignant disease beyond resection is lacking. However, due to the history of previous surgeries, the difficulty of managing malignancy after CC resection has increased. The principles of managing malignancy after CC resection should be the same as those for biliary tract cancer. Treatment methods for bile duct cancer include surgery, radiotherapy, chemotherapy, as well as targeted and immunotherapy (7–9). In the present case, the patient successively received chemotherapy, chemotherapy combined with immunotherapy, surgery, and proton therapy, and is currently undergoing maintenance treatment with durvalumab. The patient is now in a disease-free survival state.

Proton therapy is an advanced form of radiation therapy that uses high-energy protons (positively charged hydrogen nuclei) to damage the DNA of cancer cells, thereby preventing their proliferation (10). Compared to traditional X-ray radiation therapy, proton therapy has higher precision, allowing for more accurate targeting of tumors while reducing damage to surrounding healthy tissues. Proton therapy can indeed lead to a range of gastrointestinal side effects and complications (11). Proton therapy is challenging due to the close proximity of the gastrointestinal tract to the tumor. Yamazaki et al. reported that proton therapy was used to treat unresectable and/or recurrent extrahepatic biliary tract cancer and found that a narrower distance between the tumor and the digestive tract (less than 2 cm) was identified as a poor prognostic factor for overall survival (12). Therefore, to achieve a better prognosis, it is reasonable to enlarge the space between the tumor and the digestive tract. Uehara et al. reported a case of a patient with a history of distal gastrectomy and Billroth-I reconstruction for cancer, in whom a tumor in liver S3 was found0 (13). An absorbable spacer was placed, and proton therapy was performed to avoid adverse events. In the present case, we performed space-making techniques by disassembling the bilioenteric anastomosis and filling the space with a greater omentum strip to isolate the bile duct and intestines. The patient currently has almost no gastrointestinal reactions after receiving proton therapy.

## Conclusion

As CC-associated malignancy poses a lifelong risk even with complete resection, surveillance should be maintained throughout the follow-up period. Comprehensive treatment should be adopted to improve prognosis in malignancy after CC resection.

## Data Availability

The raw data supporting the conclusions of this article will be made available by the authors, without undue reservation.
